# Sarcopenia, Diet, Physical Activity and Obesity in European Middle-Aged and Older Adults: The LifeAge Study

**DOI:** 10.3390/nu13010008

**Published:** 2020-12-22

**Authors:** Pablo Jorge Marcos-Pardo, Noelia González-Gálvez, Abraham López-Vivancos, Alejandro Espeso-García, Luis Manuel Martínez-Aranda, Gemma María Gea-García, Francisco Javier Orquín-Castrillón, Ana Carbonell-Baeza, José Daniel Jiménez-García, Daniel Velázquez-Díaz, Cristina Cadenas-Sanchez, Emanuele Isidori, Chiara Fossati, Fabio Pigozzi, Lorenzo Rum, Catherine Norton, Audrey Tierney, Ilvis Äbelkalns, Agita Klempere-Sipjagina, Juris Porozovs, Heikki Hannola, Niko Niemisalo, Leo Hokka, David Jiménez-Pavón, Raquel Vaquero-Cristóbal

**Affiliations:** 1 Research Group on Health, Physical Activity, Fitness and Motor Behaviour (GISAFFCOM) and Physical Activity and Sport Sciences Department, Faculty of Sport, Catholic University San Antonio of Murcia, 30107 Murcia, Spain; pmarcos@ucam.edu (P.J.M.-P.); alvivancos@ucam.edu (A.L.-V.); aespeso@ucam.edu (A.E.-G.); lmmartinez2@ucam.edu (L.M.M.-A.); gmgea@ucam.edu (G.M.G.-G.); forquin@ucam.edu (F.J.O.-C.); rvaquero@ucam.edu (R.V.-C.); 2 Active Aging, Exercise and Health/HEALTHY-AGE Network, Consejo Superior de Deportes (CSD), Ministry of Culture and Sport of Spain, 28040 Madrid, Spain; ana.carbonell@uca.es (A.C.-B.); cristina.cadenas.sanchez@gmail.com (C.C.-S.); chiara.fossati@uniroma4.it (C.F.); fabio.pigozzi@uniroma4.it (F.P.); 3 MOVE-IT Research Group, Department of Physical Education, Faculty of Education Sciences, University of Cádiz, 11519 Cádiz, Spain; josedaniel.jimenez@uca.es (J.D.J.-G.); daniel.velazquez@uca.es (D.V.-D.); david.jimenez@uca.es (D.J.-P.); 4 Department of Movement, Human and Health Sciences University of Rome “Foro Itálico”, 00135 Roma, Italy; emanuele.isidori@uniroma4.it (E.I.); lorenzo.rum@uniroma4.it (L.R.); 5 Health Research Institute (HRI), University of Limerick, V94 T9PX Limerick, Ireland; catherine.norton@ul.ie (C.N.); audrey.tierney@ul.ie (A.T.); 6 Faculty of Pedagogy, Psychology and Art, University of Latvia, 1586 Riga, Latvia; ilvis.abelkalns@lu.lv (I.Ä.); agita.klempere-sipjagina@lu.lv (A.K.-S.); porozjur@lu.lv (J.P.); 7 Business and Services Department, Sport and Leisure, Lapland University of Applied Sciences, 96300 Rovaniemi, Finland; heikki.hannola@lapinamk.fi (H.H.); niko.niemisalo@lapinamk.fi (N.N.); leo.hokka@lapinamk.fi (L.H.)

**Keywords:** dietary habits, muscle mass, older people, physical condition, sarcopenia, sarcopenia

## Abstract

The revised European consensus defined sarcopenia as a progressive and generalized skeletal muscle disorder that is associated with an increased likelihood of adverse outcomes including falls, fractures, physical disability and mortality. The aim of this study was to determine the prevalence of sarcopenia and analyse the influence of diet, physical activity (PA) and obesity index as risk factors of each criteria of sarcopenia. A total of 629 European middle-aged and older adults were enrolled in this cross-sectional study. Anthropometrics were assessed. Self-reported PA and adherence to the Mediterranean diet were evaluated with the Global Physical Activity Questionnaire (GPAQ) and Prevention with Mediterranean Diet questionnaire (PREDIMED), respectively. The functional assessment included handgrip strength, lower body muscle strength, gait speed and agility/dynamic balance. Of the participants, 4.84% to 7.33% showed probable sarcopenia. Sarcopenia was confirmed in 1.16% to 2.93% of participants. Severe sarcopenia was shown by 0.86% to 1.49% of participants. Male; age group ≤65 years; lower body mass index (BMI); high levels of vigorous PA; and the consumption of more than one portion per day of red meat, hamburgers, sausages or cold cuts and/or preferential consumption of rabbit, chicken or turkey instead of beef, pork, hamburgers or sausages (OR = 0.126–0.454; all *p* < 0.013) resulted as protective factors, and more time of sedentary time (OR = 1.608–2.368; *p* = 0.032–0.041) resulted as a risk factor for some criteria of sarcopenia. In conclusion, age, diet, PA, and obesity can affect the risk of having low muscle strength, low muscle mass or low functional performance, factors connected with sarcopenia.

## 1. Introduction

Concurrent with the many societal and personal challenges associated with increasingly aging populations, musculoskeletal disease has become a serious public health issue. The revised European consensus defines sarcopenia as a progressive and generalized skeletal muscle disorder that is associated with an increased likelihood of adverse outcomes including falls, fractures, physical disability and mortality [1]. It is categorized firstly as an age-related decline in muscle mass and function characterized by low muscle strength (as the principal and most reliable measure of muscle function), secondly by low muscle mass (quantity) and thirdly by low physical performance, an indication of muscle quality [1,2]. Sarcopenia is closely linked to the phenotype of fragility described by Fried et al. [3]. These authors define frailty as a clinical syndrome in which at least three or more of the following criteria must be present: Unintentional weight loss, self-reported exhaustion, muscle weakness, slow walking speed and low level of physical activity. Fragility increases the likelihood of health impairment and increased disability and decreases the quality of life [4].

In fact, it is reported that sarcopenia affects about 5% to 13% of older adults (aged 60–70 years) and as many as 11% to 50% of those aged 80 years or older [5]. However, observed losses in muscle mass and function are also reported in middle-aged adults [6]. In fact, there is a decline in muscle mass of approximately 8% per decade from the age of 40 and 15% per decade from the age of 70 [7].

Previous studies have found that sarcopenia is associated with a greater risk of morbidity and mortality, exacerbated by the presence of comorbid disease [8,9]. In addition, sarcopenia increases the risk of falls and fractures [10,11], has a negative impact on the ability to carry out daily-life activities [12] and decreases the quality of life [13,14] and independence [15,16]. On the other hand, it increases the risk of developing other diseases such metabolic syndrome [17] or chronic kidney disease. Considering all of these together, sarcopenia increases the costs of the health care system due to the increased risk for hospitalization [18,19], and it increases the need for long-term care as well [20].

Due to the effect of sarcopenia on health and the importance of early and effective interventions to prevent, delay, treat or reverse this disease, previous studies have suggested methods for evaluating sarcopenia risk in clinical practice [1]. However, some difficulties are evident, such as determining which variables to measure, how best to measure variables and which cut-off points best guide diagnosis and treatment [21]. However, the European Working Group on Sarcopenia in Older People (EWGSOP) guidelines published in 2019 proposed a selection of diagnostic measures and cut-off points which can be used in clinical practice, including muscle strength, muscle mass and physical condition assessment [1]. The most commonly used methods to measure muscle mass loss are dual energy X-ray absorptiometry (DEXA) and bioelectrical impedance analysis (BIA), handgrip strength test (HG), short physical performance (SPPB) and gait speed [22,23]. The HG and time-up and go (TUG) tests are used in the diagnosis of sarcopenia [24].

Sarcopenia is a complex condition involving hormonal, biological, nutritional, anthropometric and physical activity factors [25]. It is often debated whether the incidence of sarcopenia could be influenced by sex, although it has been found that older women show higher rates of sarcopenia than middle-aged women and middle-aged and older men [25,26,27]. This relation with sex could be a consequence of the decrease in oestrogen levels from menopause, which could affect muscle mass and muscle strength [25,27], although the relative contribution of the sex hormones is difficult to establish due to multifactorial aspects that can be influential [25].

Muscle strength is a predictor of mortality and disability in older people [28]. It has been demonstrated that nutrition and physical activity can influence muscle homeostasis and prevent the loss of muscle mass [29,30]. Increasing moderate-to-vigorous physical activity (MVPA) and/or reducing sedentary behaviour (SB) may reduce the risk of sarcopenia in older adults [31].

Nutrition and levels of physical activity also influence phenotypic characteristics [15] and are associated with functional capacity [29,32]. Increased body mass index (BMI) (kg/m^2^) has been associated with decreased cardiorespiratory fitness [33], and nutritional and exercise interventions have been proposed as a way to reduce obesity/adiposity and improve physical function with short-term efficacy [34]. On the other hand, it seems that the consumption of certain protein-rich foods may be of primary relevance to well-founded dietary guidelines for the prevention of sarcopenia [35].

Being aware of the complex nature of the causes and consequences, singly and concomitantly, of factors associated with sarcopenia, further research is required to elucidate the roles of nutrition, physical activity, phenotype, sex and age. There is currently a lack of consensus on diagnostic criteria and methods [25]. Therefore, the LifeAge study was focused on lifestyle factors and their influence on health outcomes among European middle-aged and older adults. The objectives of the study were (a) to determine the prevalence of sarcopenia based on muscle strength, muscle mass and physical performance criteria; and (b) to analyse the influence of diet, physical activity and obesity index in each criteria of sarcopenia.

## 2. Materials and Methods

### 2.1. Study Design

The LifeAge (Promoting the shift sedentary Lifestyle towards active Ageing; code: 603121-EPP-1-2018-1-ES-SPO-SCP. Erasmus + Sport Programme from European Union) cross-sectional study was designed to investigate different lifestyle factors and health outcomes among European middle-aged and older adults. The study was conducted from 1 March 2019 to 31 December 2019 in the Catholic University San Antonio of Murcia (UCAM) and Cádiz University (UCA) from Spain, University of Rome “Foro Itálico” from Italy, University of Limerick from Ireland, University of Latvia from Latvia and University of Lapland from Finland. The study complied with the Strobe Statements. This study was conducted in a sports science laboratory at each of the LifeAge study’s participating universities and/or senior centres.

### 2.2. Participants

Participants were recruited through advertisements in social and senior centres, women’s centres and presentations given at local communities at the universities participating in the LifeAge study.

Ultimately, 629 participants were recruited (65.41 ± 8.54 years old). The following inclusion criteria were considered: (a) Over 50 years old, and (b) being physically independent. Exclusion criteria were: (a) Any musculoskeletal injuries or physical limitation that may have an influence on the test, (b) taking medications known to influence physical performance and (c) moderate to severe cognitive impairment diagnosed by a physician, or severe psychiatric problems (treatment due to depression or psychosis).

To establish the sample size, the Rstudio 3.15.0 software was used. Power and sample size were established with respect to the standard deviation for HG in a previous study [10]. The total sample size for this study consisted of 629 participants, which provided a power of 95% and a significance level of α = 0.05, with an estimated error of 0.37 kg reported. With regard to the distribution by country, the sample size consisted of 102 to 152 participants with an estimated error from 0.76 kg to 0.92 kg. Due to the missing values, analyses contained a total of between 583 and 629 participants, reporting an estimated error from 0.37 kg to 0.38 kg.

### 2.3. Ethics Approval and Consent to Participate

The present study obtained approval for use in a European project by the Catholic University San Antonio of Murcia ethics committee on research (code: CE031907), in accordance with the Declaration of Helsinki. Prior to participation in the study, all participants signed informed consent forms.

### 2.4. Variables and Instruments

The same trained researchers selected by each test centre performed all protocol visits which consisted in a single testing session. The participants were examined with the standardized temperature of 24 °C.

Muscle strength (muscle quality) was assessed with the handgrip strength (HG) and chair stand tests, muscle quantity was measured through muscle mass and functional performance was measured by gait speed, the timed-up-and-go-test (TUG) and the short physical performance battery (SPPB). The risk associated with each of these variables was defined in connection with the EWGSOP2 cut-off point. A low muscle strength (muscle quality) was considered with a value of less than 27 kg for men and 16 kg for women in the HG test, or more than 15 s for 5 chair stand tests. The cut-off point for low functional performance was established as equal to or less than 0.8 m/s for gait speed, equal to or less than 8 points on the SPPB battery or equal to or greater than 20 s in TUG [1]. For muscle mass, less than 28.3 kg for men and 17.7 kg for women was reported as low muscle quantity with the Lee equation, based on a previous study which established cut-offs in comparation with DEXA derived EWGSOP criteria [36].

HG was measured with the participant in an upright standing position with the arms by their sides. Each participant was asked to squeeze the grip with maximal strength for 3 s with the right hand. A mean was calculated from 3 repetitions [37]. A digital grip strength dynamometer was used for this (TKK 5401; Takei Scientific Instruments Co., Ltd., Tokyo, Japan) [1,22].

The chair stand test was used to measure the strength of the leg muscles. This test measures the time needed by the participant to move 5 times from a seated position to the standing position as quickly as possible without using the arms. Only 1 test trial was allowed [1].

Muscle mass was estimated based on the Lee equation [38], as it is the recommended muscle mass formula for middle-aged and older adults [39]. Triceps, thigh and calf skinfolds were measured with a calliper (Harpenden, London, UK), with a precision of 0.2 mm. Relaxed arm, middle-thigh and calf girths were measured with an anthropometric measuring tape (W606PM, Lufkin, EE.UU.), following the protocol from the International Society for the Advancement on Kinanthropometry (ISAK) [40].

Gait speed was assessed with a 4 m test. For this test, the person had to walk at their usual pace and the total time needed to walk 4 m was recorded. This test has reported a high predictive validity and has been used extensively [41,42,43,44,45]. With the intention of ensuring a reliable measurement, 2 photocells connected to a computer were used in the test in a subsample of the present study (MuscleLab, Ergotest, Langesund, Norway). These were placed at the beginning and end of the 4 m lane. The average value of 2 attempts was recorded.

In the TUG test, the participants had to get up from the chair, walk, go around a cone placed 3 m away, return to the chair and sit down again, without using their arms, as fast as possible without running. The best of 2 attempts was used for the analysis [29].

The SPPB battery is composed of the standing balance test, chair stand test and 4 m gait walk [46]. For balance, the participant had to maintain 3 different positions for 10 s: (a) Feet together, (b) semi-tandem position (the ankle of 1 foot behind the joint of the other foot) and (c) tandem position (ankle of 1 foot directly behind the other foot and touching it). For the chair stand test and 4 m gait walk, the same procedure was followed as explained above. Up to 4 points could be obtained in each test, assuming a score range between 0 to 12 points. A higher score indicated a better physical performance [41,46].

The International Society for Advancement on Kinanthropometry (ISAK) guidelines were followed for measuring the kinanthropometric parameters [40]. Weight (kg) was evaluated in light clothing without footwear and was measured to the nearest 0.1 kg with an electronic scale (Tanita BC-418, Illinois, MA, USA). Height (cm) was measured using a stadiometer to the nearest millimetre (Tanita HR001, Illinois, MA, USA). After that, BMI was calculated as weight (kg) divided by height (m) squared [47]. BMI was classified as normal weight (18.5–24.9 kg/m^2^), overweight (25.0–29.9 kg/m^2^), obesity class I (30.0–34.9 kg/m^2^) and obesity class II (35.0–39.9 kg/m^2^) [48].

Moderate physical activity (MPA), vigorous physical activity (VPA), MVPA and sedentary behaviour was estimated using the Global Physical Activity Questionnaire (GPAQ) [49]. This questionnaire was developed by the World Health Organization (WHO) and classifies participants by activity level, thereby allowing for the study of trends and associations with other types of behavioural or health outcomes. Participants were considered as active if they practiced at least 300 min per week of MVPA, which is the recommendation for maximizing the health benefits [50].

Adherence to the Mediterranean diet (AMedDiet) was evaluated using the PREDIMED score. This is an instrument used to assess the adherence to typical food components of the traditional Mediterranean diet [51]. The reliability of this test was verified in a validation study in Spanish people older than 65 years [52,53]. A score of 7 points or more on the questionnaire is classified as moderate-high AMedDiet, and a score of less than 7 points on the questionnaire is classified as low adherence [54].

The physical condition assessment included HG, lower body muscle strength, gait speed and agility/dynamic balance [23,55,56,57].

### 2.5. Statistical Analyses

The Kolmogorov–Smirnov test and Mauchly’s W-test were used to evaluate the normality and the sphericity of the data. Mean and standard deviation were calculated from the quantitative variables, and frequency and percent were used for the qualitative variables. VPA, MPA and sedentary time per day were categorized by 50th percentile. Logistic regression analyses were used to estimate the risk or associations between the dependent variables and each independent variable. The results were reported as raw and adjusted odds ratios (ORs) with 95% confidence intervals (CIs). Potential confounders were selected based on previously-published work [58,59]. In addition, adjustments for sex and age were performed. The 95% CI of the odds ratios was set to express the magnitude of the associations. The statistical analyses were performed using IBM SPSS Statistics (version 24.0). An error of *p* ≤ 0.05 was established.

## 3. Results

Table 1 shows the characteristics of the European middle-aged and older adult participants overall and by country. The total sample consisted of 629 participants distributed in the following countries: Spain (*n* = 145, 23.05%), Latvia (*n* = 152, 24.17%), Ireland (*n* = 127, 20.19%), Italy (*n* = 103, 16.38%) and Finland (*n* = 102, 16.22%).

Of these, 54.9% of the sample was married, 12.45% was single, 8.57% was divorced/separated and 11.22% was widowed. Arterial hypertension was reported by 21.63% of the sample, infarction by 3.45% and other heart disease by 11.69%. Varices, arthrosis, cervical pain and lumbar pain was mentioned by 12.96%, 28.24%, 10.31% and 18.49%, respectively. Allergy, asthma and chronic obstructive pulmonary disease was reported by 10.09%, 8.21% and 5.08%, respectively. Depression and anxiety were indicated by 6.34% and 3.73% of the sample, respectively.

A total of 4.82% and 7.40% of the sample showed a probable sarcopenia measurement according to the HG and chair stand tests (muscle quality indicators), respectively. Sarcopenia was confirmed in 2.93% (HG + muscle mass), and 1.16% (chair stand test + muscle mass) of the total sample (muscle quality and quantity indicators). A total between 0.86% and 1.49% of the sample showed severe sarcopenia (HG or chair stand test + muscle mass + gait speed or SPPB) (muscle quality and quantity and functional indicators) (Table 2).

The risk factors associated with probable sarcopenia (muscle strength as muscle quality factor), confirmed sarcopenia (muscle quantity) and severe sarcopenia (functional performance), and the influence of demographics, anthropometrics, physical activity and dietary habits in these risk factors is shown in Table 3, Table 4, Table 5, Table 6, Table 7, Table 8 and Table 9, unadjusted and adjusted according to sex and age.

After adjusting for age, the male sex was found to be a protective factor for functional performance (severe sarcopenia factor) measured by the gait speed test (OR = 0.464; *p* = 0.009). After adjusting for sex, an age under 65 years resulted as a protective factor for muscle strength/muscle quality (probable sarcopenia factor) measured by the HG (OR = 0.296; *p* = 0.006) and chair stand tests (OR = 0.428; *p* = 0.013) for muscle quantity measured by muscle mass (OR = 0.336; *p* < 0.001), for functional performance (severe sarcopenia factor) according to the gait speed (OR = 0.141; *p* < 0.000) and SPPB (OR = 0.129; *p* = 0.001) and having at least one criterion for sarcopenia (OR = 0.254; *p* < 0.000).

In connection with BMI, after adjusting for sex and age, normal weight + overweight (vs obesity) were considered as protective factors for muscle strength/muscle quality (probable sarcopenia factor), measured by the chair stand test (OR = 0.319; *p* < 0.000), functional performance (severe sarcopenia factor) assessed by gait speed (OR = 0.435; *p* = 0.004) and SPPB (OR = 0.193; *p* < 0.000) and having at least one criterion for sarcopenia (OR = 0.485; *p* = 0.005).

After adjustment, practicing VPA resulted as a protective factor for muscle strength/muscle quality (probable sarcopenia factor) classified with the chair stand test (OR = 0.338; *p* = 0.017), functional performance (severe sarcopenia factor) measured by gait speed (OR = 0.288; *p* = 0.001) and by having at least one criterion for sarcopenia (OR = 0.360; *p* < 0.000).

Consumption of more than one portion per day of red meat, hamburgers, sausages or cold cuts was considered as a protective factor for muscle strength/muscle quality (probable sarcopenia factor), muscle quantity (confirmed sarcopenia factor), and functional performance (severe sarcopenia factor) (chair stand test: OR = 0.375; *p* = 0.003; muscle mass: OR = 0.480; *p* < 0.001; gait speed: OR = 0.540; *p* = 0.013), having at least one criterion for sarcopenia (OR = 0.608; *p* = 0.017). When this analysis was adjusted by age and sex, only muscle strength as indicator of a muscle quality based on the chair stand test (OR = 0.443; *p* = 0.016) and muscle quantity based on muscle mass (OR = 0.466; *p* < 0.001) remained significant.

Preferential consumption of rabbit, chicken or turkey instead of beef, pork, hamburgers or sausages, after adjusting according to sex and age, was recorded as a protective factor for and functional performance (severe sarcopenia factor) measured by SPPB (OR = 0.382; *p* = 0.019). However, preferential consumption of rabbit, chicken or turkey instead of beef, pork, hamburgers or sausages, after adjusting according to sex and age, was recorded as a risk factor for muscle quantity (confirmed sarcopenia factor) measured by muscle mass (OR = 1.759; *p* = 0.011). Conversely, male sex was considered as a risk factor for muscle quantity (confirmed sarcopenia factor) measured by muscle mass (OR = 2.090; *p* < 0.001). Normal weight + overweight (vs obesity) were considered as a risk factor for muscle quantity (confirmed sarcopenia factor), measured by muscle mass (OR = 3.143; *p* < 0.001). AMedDiet was recorded as a risk factor after adjusting according to sex and age for muscle quantity (confirmed sarcopenia factor) measured by muscle mass (OR = 1.555; *p* = 0.025). The measured sedentary time over 300 min/week was considered as a risk factor for functional performance measured with the SPPB (OR = 2.243; *p* = 0.048) and by failing at least in one variable connected with sarcopenia (OR = 1.608; *p* = 0.041) after adjustment by sex and age.

In connection with age and sex, being older than 65 years showed less OR than being younger than 65 years (OR = 0.477; *p* < 0.0201), and males reported a higher OR than females (OR = 1.573; *p* = 0.010) for VPA. Older people reported a lower OR than younger people (OR = 0.654; *p* = 0.008), and females reported a lower OR than males (OR = 1.654; *p* = 0.003) for eating more than one portion of red meat, hamburgers, sausages or cold cuts. Comparing normal weight + overweight by BMI vs. obesity, normal weight + overweight participants showed high OR than obese for low muscle mass (OR = 2.763; *p* < 0.000). Obese participants reported higher value of OR than normal weight + overweight participants (OR = 1.653; *p* = 0.010).

## 4. Discussion

The focus of the present study was to determine the prevalence of sarcopenia based on muscle strength as a muscle quality indicator, muscle mass as a muscle quality indicator, and physical performance as a functionality indicator in European middle-aged and older adults. The EWGSOP established that muscle strength as a muscle quality indicator, muscle mass as a muscle quality indicator, and physical performance as a functionality indicator could be among the criteria used to diagnose sarcopenia, with this hierarchical order [1]. Probable sarcopenia was shown in 4.82% (based on the HG results as muscle quality factor) and 7.40% (based on the chair stand test results as muscle quality factor) of the total sample. Muscle strength (muscle quality indicator) has been suggested by the EWGSOP as the standard criterion to determine probable sarcopenia due to its simplicity, reliability and relatively low cost. It is, in fact, the most widely used method in clinical practice [1].

Confirmed sarcopenia was reported from 2.29% to 2.93% of the total sample. A low muscle strength (muscle quality indicator) and low muscle mass (muscle quantity indicator) have been established as criterions to determine sarcopenia in the European consensus of 2019, specifically the appendicular skeletal muscle mass, that is, the sum of the lean mass of arms and legs [1,60]. A note of caution should be stated here, because the most common methods used to determine muscle mass (magnetic resonance imaging, computed tomography, dual-energy X-ray absorptiometry, bioelectrical impedance and kinanthropometry) are all indirect methods to estimate body composition from two to five components following the tissue model, with their own inherent advantages and disadvantages [1,61,62,63]. Although these methods for assessing body composition have been demonstrated to have reliability and validity, it is not possible to compare the body composition results obtained using different methods [64]. In the current work, kinanthropometry, using standard ISAK techniques by trained observers, was used, as it provides many benefits for field assessments [65]. However, there are different formulas to estimate body composition [66], and to minimise estimation discrepancies, the Lee formula was chosen in the present research, as it has been proposed as the preferred option for middle-aged and older adults [39]. Furthermore, it includes calf girth in the formula, which has been indicated as one of the best indicators of muscle mass in older adults [1]. However, the EWGSOP has not established different cut-off points depending on the method used to estimate body composition [1], and the cut-off points considered in the current study were based on a study which compared DEXA and muscle mass with the Lee equation [36]. Furthermore, although the Lee formula uses corrected girths from the arm, thigh and leg to estimate muscle mass [39], this formula does not exactly estimate the appendicular skeletal muscle mass. Thus, these are important issues for future research.

A total of 0.86–1.49% of the total sample showed severe sarcopenia based on muscle strength (muscle quality indicator), muscle mass (muscle quantity indicator), and physical performance (functionality) results. Along this line, although physical performance as a functional performance indicator is a multidimensional concept, including muscle, central and peripheral nervous function, balance, gait disorder and dementia [1,67], gait speed is considered a fast, safe and highly reliable test for the classification of patients with a severe level of sarcopenia, and has been shown to predict adverse outcomes related to it [68,69,70,71].

Another finding was that the group aged below 65 years showed a significantly lower probability for obtaining values under the cut-off point in muscle strength as a muscle quality indicator (60.1 to 68.6% lower risk), muscle mass as a muscle quantity indicator (66.4% lower risk) and physical performance as a functional indicator (84.7% to 87.7% lower risk) than those older than 65 years. Previous studies have found a similar tendency, because muscle strength decline increases with age as a consequence of the denervation of motor units and conversion of fast type II muscle fibres into slow type I fibres [5].

The current study found that males had a 109% higher risk of sarcopenia than females based on muscle mass (muscle quantity indicator). However, sex was not found to be a determinant factor for probable sarcopenia or severe sarcopenia based on the majority of strength (muscle quality indicator) and physical tests (functional indicator indicator), respectively. In fact, males showed a 53.6% lower risk of sarcopenia than females based on gait speed results (functional indicator), and no differences were found in other tests. The controversy in scientific evidence regarding whether sarcopenia is dependent on sex has raged unabated for over a decade. On the one hand, some studies have found that the incidence in older women is higher than in middle-aged adult women and in men [7,25,26,27], and previous studies have reported that sex hormones induce changes in the quantity and the quality of the muscle mass [7]. On the other hand, physical exercise, sedentary behaviours or the obesity index could also explain the differences found in the incidence of sarcopenia between sexes [7,25]. Thus, a multifactorial approach is needed in future studies [72].

The second objective of the present study was to analyse the influence of diet, physical activity and obesity index as risk or protective factors for muscle strength as a muscle quality indicator, muscle quantity, and functional performance. Having a BMI lower than 30 kg/m^2^ was a preventative factor for low muscle strength as a muscle quality indicator (68.6% lower risk based on the chair stand test), low functional performance (55.3 to 79.9% lower risk based on gait speed test and the SPPB battery) and of failing in at least one variable associated with sarcopenia (48.6% lower risk) after adjusting according to sex and age. Researchers have shown that obesity is related to a poor physical fitness and functionality [73,74]. In fact, obesity increases the quantity of fat infiltration into muscles, decreasing the muscle’s physical function [75,76]. Consequently, the probability of suffering sarcopenia based on muscle quality and functional performance could be high. Furthermore, physical activity could modulate the relation between obesity and physical condition [74,77,78]. The findings of the present study showed that obese participants were 65.3% more probable to be sedentary, which may explain the relation between obesity and physical fitness. However, having a BMI over 30 kg/m^2^ was related to a greater probability of having a muscle mass (muscle quantity) within the range of protective values of sarcopenia (−185% lower risk). In fact, no obese middle-aged and older adults showed a high risk of having low muscle mass (176.3% higher risk). Previous studies have found a relation between IMC and muscle size of muscle in older, so high BMI acts as loading stimulus to the mass muscle [79]. From the findings of this research, it could be inferred that factors associated with the quantity and quality of muscle may be different, according to the findings of previous research [80]. Despite these promising results, questions remain.

An important clinically relevant finding was that sedentary time over 300 min/week was considered as a risk factor for low functional performance (SPPB) and having at least one criterion for sarcopenia (124.3% and 60.8% higher risk, respectively), after adjusting by sex and age. Previous studies have found that sedentary behaviour contributes to the underlying mechanisms of sarcopenia. Conversely, greater levels of activity may help to prevent, reverse or modify sarcopenia [81,82]. Furthermore, the current study found that practicing VPA (50th percentile) was a protective factor for muscle strength as a muscle quality indicator (63.6% and 73.8% lower risk in the HG and chair stand test, respectively), functional performance (62.7% lower 79% lower risk in the SPPB battery and gait speed, respectively) and having at least one criterion for sarcopenia (71.8% lower risk). However, people older than 65 years had lower VPA levels (OR = 0.477; *p* < 0.0201), and males had higher VPA levels (OR = 1.573; *p* = 0.010). Therefore, after adjusting by sex and age, differences were only found in muscle strength as a muscle quality indicator (66.2% lower risk based on the chair stand test), functional performance (71.2% lower risk in gait speed) and having at least one criterion for sarcopenia (64.0% lower risk). However, MPA based on the 50th percentile or MVPA practice based on health practice recommendations were not found to be factors that prevented the risk of either variable associated with sarcopenia in the current work. This finding was also reported by Bann et al., who found that the time spent in practicing high-intensity activities was associated with greater HG, but this was not the case for the practice of lower intensities activities [83]. Previous studies have suggested the superior effect of vigorous exercise as a health-protecting factor [84,85]. Randomized controlled trials have demonstrated that high-intensity interval training (HIIT) improved physical performance associated with strength, endurance and gait speed in older mice [86]; that low-frequency HIIT intervention improved peak muscle power in lifelong sedentary individuals [87]; and that maximal-intensity isokinetic eccentric resistance HIIT can generate higher muscle adaptations without negative impacts on muscle function [88]. This observation may support the hypothesis that reducing sedentary time is important, but simply meeting the recommended levels of physical activity is not enough to prevent sarcopenia, and engaging in vigorous activity every week is also needed.

Nutrition has been recognised as a key factor in promoting healthy lifestyles in the growing older population across Europe [89]. Surprisingly, AMedDiet was associated with a higher risk factor of sarcopenia based on muscle quantity (muscle mass) (55.55% lower risk), while there was no significant association between AMedDiet and parameters related to muscle quality and functionality. A possible explanation for this might be that AMedDiet recommends consuming red meat and processed meet less than two or one times per week [90]. The present study found that the consumption of more than one portion per day of red meat, hamburgers, sausages or cold cuts was a protective factor for low muscle strength as a muscle quality indicator (62.5% based on the chair stand test), muscle quantity (52.0% lower risk based on muscle mass), functional performance (45% lower risk based on the gait speed test) and having at least one criterion for sarcopenia (39.2% lower risk). However, older people consumed red meat, hamburgers, sausage or cold with less frequency (OR = 0.654; *p* = 0.008) and females consumed red meat, hamburgers, sausages or cold cuts with less frequency (OR = 1.654; *p* = 0.003), so after adjusting the results by age and sex, 55.7% lower risk was found for muscle strength as muscle quality indicator according to the chair stand test and 53.4% lower risk based on muscle quantity measured by muscle mass. Furthermore, the preferential consumption of rabbit, chicken or turkey instead of beef, pork, hamburgers or sausages was recorded as a protective factor for physical performance (functional indicator) according to the SPPB battery (63.9% lower risk) after adjusting according to sex and age. This finding reflects the well-founded dietary tenet that consumption of protein-rich foods with an appropriate quantity, quality, meal frequency and timing relative to a training stimulus favours muscle accretion and may prevent muscle protein degradation [35]. In fact, meat contains leucine, an essential amino acid which activates the signalling pathways leading to protein synthesis [91]. It has been found that a high proportion of leucine is required in order to reverse the suboptimal muscle protein synthesis and increase lean mass in older adults [92,93]. Previous studies have also found that protein intake is a factor that correlates with the diminished muscle strength [94], and that leucine as protein supplements can increase skeletal muscle synthesis of older mice [95] and the anabolic response in older men [96]. It is possible, therefore, that intentional consumption of protein food groups may attenuate the risk of factors connected with sarcopenia.

However, no significant influences of other protein-rich foods, such as legumes or fish and/or shellfish, were found. Although this kind of food could have similar proprieties as meat in preventing sarcopenia [94], a high percentage of the European middle-aged and older adults included in the present study did not meet the recommendations for the intake of fish or shellfish and legumes per week, which can explain the lack of a correlation with sarcopenia. These results are in line with those from previous studies, which found that older adults did not meet the recommended legume intake per week in the European population [89], likely attributable to cultural habits and other preferences of food in developed countries [97]. On the other hand, previous studies have found that a high price is a perceived barrier to meet the guidelines for the intake of fish, especially in lower socioeconomic status older adults [98]. Further work is required to establish the preventive factor of sarcopenia with interventions based on increasing the intake of fish, shellfish and legumes.

Surprisingly, only one older adult showed a low physical performance based on the time-up-and-go test. This test has been proposed by the EWGSOP, just as the other tests, and batteries were included in the present study as a measure of functionality [1]. However, due to differences in the findings shown by the time-up-and-go test and the others, it is possible that cut-off points for this physical condition test, related to a low or high physical performance, need to be reviewed. Along the same line, as a consequence of the differences in muscle mass estimation depending on the method, model and/or formula [62,63,64,65,66], cut-off points are needed for each option. These are important issues for future research.

Concerning the study limitations, this study did not include some variables that could also influence sarcopenia, such as smoking [99], genetics [100], malnutrition and chronic inflammation [101], among others. Other possible sources of bias could be that the included population was relatively healthy and active. The results could be different if the characteristics of the population change. Furthermore, the physical activity was measured with a questionnaire and not with an objective system such as an accelerometer. Furthermore, the kind of physical activity performed was not recorded, and some specific training methods, such as resistance training, could influence sarcopenia [94]. Finally, a method to estimate muscle mass, but not appendicular muscle mass, was utilised, so the muscle mass results should be replicated with other methods of assessing muscle mass to confirm the results of this study.

## 5. Conclusions

European middle-aged and older adults showed probable sarcopenia ranging from 4.82% to 7.40% based on muscle strength as a muscle quality indicator, while the percentage of sarcopenia cases was very low. In general, being older; obese; sedentary; not performing vigorous physical activity; eating less than 100–150 g of red meat, hamburgers, sausages or cold cuts; or eating more red meat, hamburgers, sausages or cold cuts than rabbit/chicken/turkey resulted in an increased risk of having low muscle strength (muscle quality indicator), low muscle mass (muscle quantity indicator) or low functional performance, factors connected with sarcopenia.

Physicians, nutritionists and physical educators play an important role in the assessment, management and prevention of sarcopenia to reduce its impact on the utilisation of health care resources. The coordination between these multidisciplinary teams is suggested for the correct prescription of an exercise program aimed at European middle-aged and older adults that includes moderate- and high-intensity resistance training as well as nutritional education. In addition, these scientists and public health authorities should collaborate to find solutions for educating toward a healthy lifestyle for a successful and active ageing.

## Figures and Tables

**Table 1:** Characteristic of European middle and older adults, overall and by country [%(*n*)—X ± SD].

	All (*n* = 629)	Spain (*n* = 145)	Latvia (*n* = 152)	Ireland (*n* = 127)	Italy (*n* = 103)	Finland (*n* = 102)
Male sex (vs. women)	37.20 (234)	40.00 (58)	45.39 (69)	29.13 (37)	42.72 (44)	25.49 (26)
Age	65.41 ± 8.54	66.04 ± 9.58	64.00 ± 9.96	66.25 ± 6.56	64.08 ± 8.30	66.94 ± 6.54
≤65 years old (vs. >65 years old)	47.69 (300)	42.76 (62)	55.92 (85)	44.09 (56)	53.40 (55)	41.18 (42)
BMI (kg/m^2^)	27.02 ± 4.46	28.52 ± 3.71	27.13 ± 4.96	26.98 ± 4.84	25.96 ± 3.56	25.86 ± 4.43
Normal-weight or overweight (vs. obese)	78.22 (492)	69.66 (101)	76.32 (116)	76.38 (97)	89.32 (92)	84.31 (86)
MPA over 50th percentile (vs. less than 50th percentile)	49.11 (303)	46.53 (67)	50.00 (75)	59.50 (72)	41.58 (42)	46.53 (47)
VPA over 50th percentile (vs. less than 50th percentile)	35.43 (214)	27.08 (39)	26.17 (39)	48.72 (57)	25.74 (26)	56.99 (53)
MVPA over recommendation of 300 min/week (vs. less than)	78.25 (464)	82.52 (118)	72.79 (107)	81.98 (91)	71.72 (71)	82.80 (77)
Sedentary activity over 50th percentile (vs. less than 50th percentile)	41.06 (255)	54.48 (79)	55.70 (83)	27.56 (35)	43.69 (45)	13.40 (13)
Low muscle strength by HG (vs. high)	4.82 (30)	8.97 (13)	4.64 (7)	5.69 (7)	2.91 (3)	0.00 (0)
Low muscle strength by chair stand test (vs. high)	7.40 (46)	15.49 (22)	6.04 (9)	0.79 (1)	6.86 (7)	6.86 (7)
Low muscle quantity by muscle mass (vs. high)	27.63 (171)	40.28 (58)	5.37 (8)	31.97 (39)	29.41 (30)	35.29 (36)
Low functional performance by gait speed (vs. high)	13.57 (84)	20.86 (29)	18.00 (27)	1.57 (2)	20.39 (21)	5.00 (5)
Low functional performance by TUG (vs. high)	0.16 (1)	0.70 (1)	0.00 (0)	0.00 (0)	0.00 (0)	0.00 (0)
Low functional performance by SPPB (vs. high)	4.70 (28)	8.89 (12)	9.52 (14)	9.52 (14)	1.96 (2)	0.00 (0)
Have at least one criterion for sarcopenia by European Consensus	20.24 (117)	29.32 (39)	21.83 (31)	10.28 (11)	25.00 (25)	11.46 (11)
AMedDiet (vs. no AMedDiet)	34.45 (216)	69.44 (100)	9.21 (14)	26.77 (34)	45.63 (47)	20.79 (21)
>1 portion/day of red meat, hamburgers, sausages or cold cuts (portion: 100–150 g) (vs. <1 portion)	51.83 (325)	20.83 (30)	77.63 (118)	65.35 (83)	39.81 (41)	52.48 (53)
≥3 portion of legumes per week (portion: 150 g) (vs. <3 portion)	21.05 (132)	34.72 (50)	7.24 (11)	30.71 (39)	25.24 (26)	5.94 (6)
≥3 portion of fish or shellfish per week (portion: 100–150 g of fish; 200 g of Shellfish) (vs. <3 portion)	24.08 (151)	46.53 (67)	7.24 (11)	24.41 (31)	15.53 (16)	25.74 (26)
Preferential consumption of rabbit/chicken/turkey instead of beef/pork/hamburgers/sausages (vs. no preferential)	72.25 (453)	81.94 (118)	54.61 (83)	78.74 (100)	66.02 (68)	83.17 (84)

% = percentage; AMedDiet = adherence to Mediterranean diet; BMI = body mass index; DS = standard deviation; g = grams; HG = handgrip strength; kg = kilograms; m = meter; MPA = moderate physical activity; MVPA = moderate to vigorous physical activity; *n* = number of sample; SPPB = short physical performance battery; TUG = timed-up and go test; VPA = vigorous physical activity; X = mean.

**Table 2:** Probable sarcopenia, confirmed sarcopenia, and severe sarcopenia of the total of the sample and for countries [%(*n*)].

	Probable Sarcopenia		Confirmed Sarcopenia		Severe Sarcopenia					
	HG	Chair Stand Test	HG + Muscle Mass	Chair Stand Test + Muscle Mass	HG + Muscle Mass + Gait Speed	HG + Muscle Mass + TUG	HG + Muscle Mass + SPPB	Chair Stand Test + Muscle Mass + Gait Speed	Chair Stand Test + Muscle Mass + TUG	Chair Stand Test + Muscle Mass + SPPB
Total	4.82(30)	7.40 (46)	2.30 (18)	2.29 (14)	1.16 (7)	0.00 (0)	0.86 (5)	1.49 (9)	0.00 (0)	0.86 (5)
Spain	8.97(13)	15.49(22)	7.64 (11)	7.80 (11)	5.07 (7)	0.00 (0)	3.73 (5)	5.88 (8)	0.00 (0)	3.76 (5)
Latvia	4.64 (7)	6.04 (9)	0.68 (1)	0.00 (0)	0.00 (0)	0.00 (0)	0.00 (0)	0.00 (0)	0.00 (0)	0.00 (0)
Ireland	5.69 (7)	0.79 (1)	3.36 (4)	0.00 (0)	0.00 (0)	0.00 (0)	0.00 (0)	0.00 (0)	0.00 (0)	0.00 (0)
Italy	2.91 (3)	6.86 (7)	1.96 (2)	0.00 (0)	0.00 (0)	0.00 (0)	0.00 (0)	0.00 (0)	0.00 (0)	0.00 (0)
Finland	0.00 (0)	6.86 (7)	0.00 (0)	2.94 (3)	0.00 (0)	0.00 (0)	0.00 (0)	1.00 (1)	0.00 (0)	0.00 (0)

HG = handgrip strength; SPPB = short physical performance battery; TUG = timed-up and go test.

**Table 3:** Odds ratio of muscle strength by HG unadjusted and adjusted by sex and age.

	HG Low Muscle Strength % (*n*)	HG High Muscle Strength % (*n*)	Unadjusted		Adjusted	
			OR	*p*	OR	*p*
Male sex (vs. female)	43.30 (13)	37.10 (220)	1.297	0.492	1.445	0.342
≤65 years old (vs. >65 years old)	23.33 (7)	49.24 (292)	0.314	0.008	0.295	0.006
Normal weight + overweight by BMI (vs obesity)	80.00 (24)	77.91 (462)	1.134	0.787	1.267	0.622
MPA over 50th percentile (vs. less than 50th percentile)	36.67 (11)	49.74 (289)	0.585	0.167	0.560	0.143
VPA over 50th percentile (vs. less than 50th percentile)	17.24 (5)	36.38 (207)	0.364	0.043	0.485	0.159
MVPA over recommendation of 300 min/week (vs. less than recommendation)	86.21 (25)	90.32 (504)	0.609	0.231	.538	0.157
Sedentary activity over 50th percentile (vs. less than 50th percentile)	50.00 (15)	40.85 (239)	1.448	0.323	1.727	0.158
AMedDiet (vs. no AMedDiet)	36.67 (11)	34.52 (204)	1.098	0.809	1.206	0.639
>1 portion/day of red meat, hamburgers, sausages or cold cuts (portion: 100–150 g) (vs. <1 portion)	43.33 (13)	52.12 (308)	0.703	0.350	0.838	0.651
≥3 portion of legumes per week (portion: 150 g) (vs. <3 portion)	10.00 (3)	21.32 (126)	0.410	0.148	0.449	0.199
≥3 portion of fish or shellfish per week (portion: 100–150 g of fish; 200 g of Shellfish) (vs. <3 portion)	36.67 (11)	23.35 (138)	1.9	0.101	1.77	0.157
Preferential consumption of rabbit/chicken/turkey instead of beef/pork/hamburgers/sausages (vs. no preferential)	66.67 (20)	72.59 (429)	0.481	0.755	0.917	0.832

% = percentage; AMedDiet = adherence to Mediterranean diet; BMI = body mass index; g = grams; min = minutes; HG = handgrip strength; MPA = moderate physical activity; MVPA = moderate to vigorous physical activity; *n* = number of sample; OR = odd ratio; *p* = significance; VPA = vigorous physical activity; X = mean.

**Table 4:** Odds ratio of muscle strength by chair stand test unadjusted and adjusted by sex and age.

	Chair Stand Test Low Muscle Strength % (*n*)	Chair Stand Test High Muscle Strength % (*n*)	Unadjusted		Adjusted	
			OR	*p*	OR	*p*
Male sex (vs. female)	23.91 (11)	38.72 (223)	0.498	0.05	0.524	0.075
≤65 years old (vs. >65 years old)	28.26 (13)	49.65 (286)	0.399	0.007	0.428	0.013
Normal weight + overweight by BMI (vs obesity)	56.52 (26)	80.56 (464)	0.314	0.000	0.319	0.000
MPA over 50th percentile (vs. less than 50th percentile)	42.22 (19)	49.91 (282)	0.733	0.322	0.656	0.188
VPA over 50th percentile (vs. less than 50th percentile)	13.64 (6)	37.61 (208)	0.262	0.003	0.338	0.017
MVPA over recommendation of 300 min/week (vs. less than recommendation)	90.91 (40)	90.41 (490)	0.926	0.837	0.883	0.748
Sedentary activity over 50th percentile (vs. less than 50th percentile)	44.44 (20)	40.70 (232)	1.166	0.623	1.45	0.246
AMedDiet (vs. no AMedDiet)	41.30 (19)	33.97 (195)	1.368	0.316	1.54	0.179
>1 portion/day of red meat, hamburgers, sausages or cold cuts (portion: 100–150 g) (vs. <1 portion)	30.43 (14)	53.83 (309)	0.375	0.003	0.443	0.016
≥3 portion of legumes per week (portion: 150 g) (vs. <3 portion)	17.39 (8)	21.60 (124)	0.764	0.503	0.853	0.698
≥3 portion of fish or shellfish per week (portion: 100–150 g of fish; 200 g of Shellfish) (vs. <3 portion)	32.61 (15)	23.34 (134)	1.589	0.160	1.44	0.276
Preferential consumption of rabbit/chicken/turkey instead of beef/pork/hamburgers/sausages (vs. no preferential)	76.09 (35)	71.95 (413)	1.24	0.547	1.35	0.418

% = percentage; AMedDiet = adherence to Mediterranean diet; BMI = body mass index; g = grams; min = minutes; MPA = moderate physical activity; MVPA = moderate to vigorous physical activity; *n* = number of sample; OR = odd ratio; *p* = significance; VPA = vigorous physical activity; X = mean.

**Table 5:** Odds ratio of muscle quantity by muscle mass unadjusted and adjusted by sex and age.

	Muscle Mass Low Muscle Quantity % (*n*)	Muscle Mass High Muscle Quantity % (*n*)	Unadjusted		Adjusted	
			OR	*p*	OR	*p*
Male sex (vs. female)	47.95 (82)	33.93 (152)	1.794	0.001	2.090	<0.001
≤65 years old (vs. >65 years old)	29.24 (50)	55.13 (247)	0.336	<0.001	0.290	<0.001
Normal weight + overweight by BMI (vs. obesity)	88.89 (152)	74.33 (333)	2.763	<0.001	3.143	<0.001
MPA over 50th percentile (vs. less than 50th percentile)	50.89 (86)	48.86 (214)	1.085	0.654	1.081	0.682
VPA over 50th percentile (vs. less than 50th percentile)	34.97 (57)	35.96 (155)	0.958	0.822	1.142	0.519
MVPA over recommendation of 300 min/week (vs. less than recommendation)	82.72 (134)	76.48 (322)	1.471	0.104	1.334	0.246
Sedentary activity over 50th percentile (vs. less than 50th percentile)	38.24 (65)	41.95 (185)	0.857	0.403	0.960	0.837
AMedDiet (vs. no AMedDiet)	39.77 (68)	32.96 (147)	1.343	0.113	1.555	0.025
>1 portion/day of red meat, hamburgers, sausages or cold cuts (portion: 100–150 g) (vs. <1 portion)	38.60 (66)	56.73 (253)	0.480	<0.001	0.466	<0.001
≥3 portion of legumes per week (portion: 150 g) (vs. <3 portion)	23.98 (41)	19.96 (89)	1.265	0.274	1.361	0.172
≥3 portion of fish or shellfish per week (portion: 100–150 g of fish; 200 g of Shellfish) (vs. <3 portion)	29.24 (50)	22.20 (99)	1.448	0.068	1.463	0.075
Preferential consumption of rabbit/chicken/turkey instead of beef/pork/hamburgers/sausages (vs. no preferential)	76.02 (130)	70.63 (315)	1.319	0.182	1.759	0.011

% = percentage; AMedDiet = adherence to Mediterranean diet; BMI = body mass index; g = grams; min = minutes; MPA = moderate physical activity; MVPA = moderate to vigorous physical activity; *n* = number of sample; OR = odd ratio; *p* = significance; VPA = vigorous physical activity; X = mean.

**Table 6:** Odds ratio of physical performance by gait speed unadjusted and adjusted by sex and age.

	Gait Speed Low Physical Performance % (*n*)	Gait Speed High Physical Performance % (*n*)	Unadjusted		Adjusted	
			OR	*p*	OR	*p*
Male sex (vs. female)	23.81 (20)	38.81 (213)	0.472	0.006	0.464	0.009
≤65 years old (vs. >65 years old)	13.00 (11)	53.08 (284)	0.133	<0.001	0.141	<0.001
Normal weight + overweight by BMI (vs. obesity)	65.48 (55)	80.93 (433)	0.447	0.002	0.435	0.004
MPA over 50th percentile (vs. less than 50th percentile)	48.78 (40)	49.14 (258)	0.986	0.951	0.887	0.645
VPA over 50th percentile (vs. less than 50th percentile)	12.05 (10)	39.53 (202)	0.210	<0.001	0.288	0.001
MVPA over recommendation of 300 min/week (vs. less than recommendation)	91.46 (75)	90.22 (452)	0.977	0.936	0.974	0.937
Sedentary activity over 50th percentile (vs. less than 50th percentile)	41.46 (34)	40.57 (215)	1.038	0.878	1.49	0.134
AMedDiet (vs. no AMedDiet)	34.52 (29)	34.52 (184)	1	1	1.151	0.604
>1 portion/day of red meat, hamburgers, sausages or cold cuts (portion: 100–150 g) (vs. <1 portion)	39.29 (33)	54.03 (288)	0.550	0.013	0.726	0.222
≥3 portion of legumes per week (portion: 150 g) (vs. <3 portion)	14.29 (12)	22.51 (120)	0.574	0.091	0.647	0.219
≥3 portion of fish or shellfish per week (portion: 100–150 g of fish; 200 g of Shellfish) (vs. <3 portion)	20.24 (17)	24.77 (132)	0.771	0.369	0.611	0.120
Preferential consumption of rabbit/chicken/turkey instead of beef/pork/hamburgers/sausages (vs. no preferential)	61.90 (52)	73.92 (394)	0.573	0.023	0.584	0.051

% = percentage; AMedDiet = adherence to Mediterranean diet; BMI = body mass index; g = grams; min = minutes; MPA = moderate physical activity; MVPA = moderate to vigorous physical activity; *n* = number of sample; OR = odd ratio; *p* = significance; VPA = vigorous physical activity; X = mean.

**Table 7:** Odds ratio of physical performance by SPPB unadjusted and adjusted by sex and age.

	SPPB Low Physical Performance % (*n*)	SPPB High Physical Performance % (*n*)	Unadjusted		Adjusted	
			OR	*p*	OR	*p*
Male sex (vs. female)	25.00 (7)	38.90 (221)	0.523	0.146	0.543	0.182
≤65 years old (vs. >65 years old)	10.71 (3)	49.47 (281)	0.123	0.001	0.129	0.001
Normal weight + overweight by BMI (vs. obesity)	46.43 (13)	81.16 (461)	0.201	<0.001	0.193	<0.001
MPA over 50th percentile (vs. less than 50th percentile)	42.86 (12)	49.73 (277)	0.758	0.479	0.695	0.369
VPA over 50th percentile (vs. less than 50th percentile)	17.86 (5)	36.83 (200)	0.373	0.049	0.574	0.284
MVPA over recommendation of 300 min/week (vs. less than recommendation)	92.86 (26)	90.62 (483)	1.252	0.657	1.256	0.666
Sedentary activity over 50th percentile (vs. less than 50th percentile)	53.57 (15)	40.39 (227)	1.703	0.171	2.243	0.048
AMedDiet (vs. no AMedDiet)	17.86 (5)	35.34 (200)	0.398	0.066	0.452	0.121
>1 portion/day of red meat, hamburgers, sausages or cold cuts (portion: 100–150 g) (vs. <1 portion)	50.00 (14)	52.12 (295)	0.919	0.827	1.229	0.610
≥3 portion of legumes per week (portion: 150 g) (vs. <3 portion)	14.29 (4)	21.73 (123)	0.600	0.353	0.681	0.497
≥3 portion of fish or shellfish per week (portion: 100–150 g of fish; 200 g of Shellfish) (vs. <3 portion)	21.43 (6)	24.20 (137)	0.854	0.738	0.779	0.606
Preferential consumption of rabbit/chicken/turkey instead of beef/pork/hamburgers/sausages (vs. no preferential)	50.00 (14)	73.50 (416)	0.361	0.009	0.382	0.019

% = percentage; AMedDiet = adherence to Mediterranean diet; BMI = body mass index; g = grams; min = minutes; MPA = moderate physical activity; MVPA = moderate to vigorous physical activity; *n* = number of sample; OR = odd ratio; *p* = significance; VPA = vigorous physical activity; X = mean.

**Table 8:** Odds ratio of physical performance by TUG unadjusted and adjusted by sex and age.

	TUG Low Physical Performance % (*n*)	TUG High Physical Performance % (*n*)	Unadjusted		Adjusted	
			OR	*p*	OR	*p*
Male sex (vs. female)	0.00 (0)	37.77 (230)	0.000	0.995	<0.001	0.995
≤65 years old (vs. >65 years old)	0.00 (0)	47.95 (292)	0.000	0.995	<0.001	0.994
Normal weight + overweight by BMI (vs. obesity)	0.00 (0)	78.98 (481)	0.000	0.993	<0.001	0.992
MPA over 50th percentile (vs. less than 50th percentile)	100 (1)	49.08 (293)	5,513,566.011	0.995	3,968,179.448	0.994
VPA over 50th percentile (vs. less than 50th percentile)	0.00 (0)	35.62 (208)	0.000	0.996	<0.001	0.995
MVPA over recommendation of 300 min/week (vs. less than recommendation)	100 (1)	90.40 (518)	3,605,970.628	0.997	2,913,258.847	0.996
Sedentary activity over 50th percentile (vs. less than 50th percentile)	100 (1)	41.20 (248)	6,514,011.473	0.994	6,871,803.885	0.993
AMedDiet (vs. no AMedDiet)	0.00 (0)	34.60 (210)	0.000	0.996	<0.001	0.995
>1 portion/day of red meat, hamburgers, sausages or cold cuts (portion: 100–150 g) (vs. <1 portion)	100 (1)	51.73 (314)	5,144,824.353	0.995	5,842,516.823	0.994
≥3 portion of legumes per week (portion: 150 g) (vs. <3 portion)	0.00 (0)	20.92 (127)	0.000	0.997	<0.001	0.996
≥3 portion of fish or shellfish per week (portion: 100–150 g of fish; 200 g of Shellfish) (vs. <3 portion)	100 (1)	24.05 (146)	11,064,896.16	0.993	7,259,462.894	0.992
Preferential consumption of rabbit/chicken/turkey instead of beef/pork/hamburgers/sausages (vs. no preferential)	0.00 (0)	72.65 (441)	0.000	0.993	<0.001	0.993

% = percentage; AMedDiet = adherence to Mediterranean diet; BMI = body mass index; g = grams; min = minutes; MPA = moderate physical activity; MVPA = moderate to vigorous physical activity; *n* = number of sample; OR = odd ratio; *p* = significance; TUG = time-up and go test; VPA = vigorous physical activity; X = mean.

**Table 9:** Odds ratio of have at least one criterion for sarcopenia by European Consensus unadjusted and adjusted by sex and age.

	Have at Least 1 Criterion % (*n*)	Do Not Have a Criterion % (*n*)	Unadjusted		Adjusted	
			OR	*p*	OR	*p*
Male sex (vs. female)	31.60 (37)	41.02 (190)	0.660	0.059	0.693	0.121
≤65 years old (vs. >65 years old)	23.08 (27)	54.9 (253)	0.247	<0.001	0.254	<0.001
Normal weight + overweight by BMI (vs. obesity)	70.10 (82)	82.00 (378)	0.514	0.005	0.485	0.005
MPA over 50th percentile (vs. less than 50th percentile)	48.70 (56)	50.00 (226)	0.949	0.803	0.832	0.415
VPA over 50th percentile (vs. less than 50th percentile)	16.67 (19)	41.46 (182)	0.282	<0.001	0.360	<0.001
MVPA over recommendation of 300 min/week (vs. less than recommendation)	90.27 (102)	90.47 (389)	1.080	0.769	0.953	0.867
Sedentary activity over 50th percentile (vs. less than 50th percentile)	43.97 (51)	40.57 (185)	1.149	0.507	1.608	0.041
AMedDiet (vs. no AMedDiet)	32.50 (38)	35.73 (164)	0.865	0.511	0.997	0.923
>1 portion/day of red meat, hamburgers, sausages or cold cuts (portion: 100–150 g) (vs. <1 portion)	41.90 (49)	54.25 (249)	0.608	0.017	0.737	0.176
≥3 portion of legumes per week (portion: 150 g) (vs. <3 portion)	16.20 (19)	22.66 (104)	0.662	0.132	0.731	0.283
≥3 portion of fish or shellfish per week (portion: 100–150 g of fish; 200 g of Shellfish) (vs. <3 portion)	20.50 (24)	25.05 (115)	0.772	0.306	0.671	0.139
Preferential consumption of rabbit/chicken/turkey instead of beef/pork/hamburgers/sausages (vs. no preferential)	68.40 (80)	73.42 (337)	0.783	0.277	0.872	0.582

% = percentage; AMedDiet = adherence to Mediterranean diet; BMI = body mass index; g = grams; min = minutes; MPA = moderate physical activity; MVPA = moderate to vigorous physical activity; *n* = number of sample; OR = odd ratio; *p* = significance; VPA = vigorous physical activity; X = mean.
